# Measuring home environments across cultures: Invariance of the HOME scale across eight international sites from the MAL-ED study^☆^

**DOI:** 10.1016/j.jsp.2017.06.001

**Published:** 2017-10

**Authors:** Paul C. Jones, Laura L. Pendergast, Barbara A. Schaefer, Muneera Rasheed, Erling Svensen, Rebecca Scharf, Rita Shrestha, Angelina Maphula, Reeba Roshan, Zeba Rasmussen, Jessica C. Seidman, Laura E. Murray-Kolb, The MAL-ED Network Investigators

**Affiliations:** a Temple University, Philadelphia, PA, USA; b The Pennsylvania State University, State College, PA, USA; c Aga Khan University, Pakistan; d University of Bergen, Norway; e Haydom Lutheran Hospital, Tanzania; f University of Virginia; g Institute of Medicine, Tribuhvan University, Kathmandu, Nepal; h University of Venda, South Africa; i Christian Medical College, Vellore, India; j Fogarty International Center/National Institutes of Health, Bethesda, MD, USA

**Keywords:** Measurement invariance, Validity, Culture, MAL-ED, Environment, Low-income countries

## Abstract

The home environment provides the context for much of a child's early development. Examples of important aspects of the home environment include safety, cleanliness, and opportunities for cognitive stimulation. This study sought to examine the psychometric properties of an adapted form of the Home Observation for the Measurement of the Environment (HOME; Caldwell & Bradley, 1984, 2003) across the eight international sites of the MAL-ED project (Dhaka, Bangladesh; Vellore, India; Bhakatapur, Nepal; Naushahro Feroze, Pakistan; Fortaleza, Brazil; Loreto, Peru; Venda, South Africa; Haydom, Tanzania), to identify a factor structure that fit the data at all sites, and to derive a subset of items that could be used to examine home environmental characteristics across sites. A three-factor structure (i.e., Emotional and Verbal Responsivity; Clean and Safe Environment; Child Cleanliness) was identified, and partial measurement equivalence/invariance across sites was supported. Overall, these findings lend support for the use of portions of this abbreviated and adapted version of the HOME for use among heterogeneous, cross-cultural groups in low- and middle-income nations.

## 1 Introduction

In recent years, school psychologists have been serving increasingly diverse populations within the U. S. and internationally (Jimerson et al., 2009, Mendes et al., 2014). To better serve such diverse populations, in the United States and worldwide, a comprehensive understanding of the context in which children grow and develop is necessary. School psychologists need to be aware of the connection between physical health and psychological health, and how the context of early childhood may influence later development. Experiences of early childhood, such as early exposure to toxins (Jusko et al., 2008) and parenting practices (Bradley, Corwyn, Burchinal, McAdoo, & Garcia Coll, 2001), have a significant influence on cognitive development and academic outcomes (Andrade et al., 2005, Bradley and Corwyn, 2002, Leventhal and Brooks-Gunn, 2000, Pendergast and Kaplan, 2015, Shaw, 2004, Shonkoff et al., 2012). School psychology researchers, particularly those working in low-income settings, may require a psychometrically robust measurement tool to evaluate environmental characteristics. Thus, psychometrically supported tools are necessary to ensure that early childhood development is being measured accurately across contexts.

Like most individual differences, child development varies significantly based on factors such as family values, the physical environment, or even the political structure of the community or nation. Macrosystem-level factors, such as sociocultural norms, may differentially influence home environment between cultural groups. From an ecological perspective (e.g., Bronfenbrenner, 1977), evaluation of a child's home environment may be useful as it relates to early childhood health and development. For researchers to understand the complex ways in which biological and psychosocial factors influence child development from an ecological perspective, it is also necessary to understand the environment in which the child develops – which requires the measurement of characteristics of the home environment. Moreover, a number of psychometric issues, including linguistic and cultural non-congruence, can arise when using assessments across different cultural groups (Bracken and Barona, 1991, Van De Vijver and Poortinga, 1982). Thus, systematic and psychometrically robust measurement tools are required to adequately evaluate factors relevant to cognitive, psychological, and physical development, with respect to cultural differences. The current research is an effort to provide psychometric support for the use of a home environment observation tool across multiple cultural groups.

The Home Observation for Measurement of the Environment Scale (HOME; Caldwell and Bradley, 1984, Caldwell and Bradley, 2003) is an observational tool intended to assess characteristics of the home environment that are relevant to childhood health and development, including both physical and relational dimensions. The HOME has been used in medical and epidemiological studies worldwide (e.g., Bradley, Mundfrom, Whiteside, Casey, & Barrett (1994); Bradley, Whiteside et al. (1994); Bradley et al., 1996, Walkowiak et al., 2001, Williams et al., 2003, Black et al., 2004), but studies of the psychometric properties of the HOME across cultures are limited. Measures do not always operate similarly across cultures, and it is necessary to examine whether the constructs and items function in a similar fashion across sites (e.g., measurement equivalence/invariance; Pendergast, von der Embse, Kilgus, & Eklund, 2017). To that end, the version of the HOME adapted by Black et al. (2004) was administered and the psychometric properties examined as part a larger multinational study examining several factors related to child development across each of eight sites: Dhaka, Bangladesh; Vellore, India; Bhakatapur, Nepal; Naushahro Feroze, Pakistan; Fortaleza, Brazil; Loreto, Peru; Venda, South Africa; Haydom, Tanzania.

### 1.1 Environment and child development

Early life experiences play a critical role in child development and can provide the foundation for future health and well-being (National Research Council and Institute of Medicine, 2000, Shonkoff et al., 2009, Shonkoff et al., 2012). Bronfenbrenner (1977) emphasized the importance of understanding human development in the context of the environment in which an individual exists. Accordingly, school psychologists and others working in health care and education have been encouraged to adopt an ecological perspective and consider multiple characteristics of a child's environment in research and in practice (Pendergast and Kaplan, 2015, Sheridan and Gutkin, 2000). Further, it is important to consider the role that both physical and interpersonal/social-emotional factors can play in child development.

For instance, Andrade et al. (2005) reported on the differential impact of quality household stimulation on cognitive development. Further, the effects of high levels of early stress and adversity in childhood on later development have also been well documented (e.g., Shonkoff et al., 2012). Adverse neurobiological effects can have devastating effects on children who are exposed to toxins in the home environment (e.g., Grandjean & Landrigan, 2006). Even low levels of lead exposure can have devastating effects on intellectual ability (e.g., Lanphear et al., 2005). These early childhood experiences can have significant implications for a child's later ability to learn and engage in a school environment.

There are many physical, interpersonal, and experiential factors that characterize a high-quality home environment. Factors that are commonly discussed in the psychological and educational literatures include availability of learning resources (e.g., toys and books), sensitive and responsive interactions from caregivers, and the provision of a variety of experiences to a developing child (e.g., Andrade et al., 2005). However, physical characteristics of the home environment, such as safety (e.g., Delgado et al., 2002), cleanliness (e.g., Aiello & Larson, 2002), home crowding (e.g., Solari & Mare, 2012), likelihood of exposure to neurotoxins (e.g., Grandjean & Landrigan, 2006), and likelihood of exposure to illness-causing pathogens (e.g., Cardoso, Cousens, de Góes Siqueira, Alves, & D'Angelo, 2004) also play a key role in childhood development. Quality home environments can serve as protective factors against adverse outcomes (e.g., Bradley, Whiteside et al., 1994).

#### 1.1.1 Relational environmental characteristics (parental responsiveness)

Andrade et al. (2005) examined the effect of maternal level of education on the quality of cognitive stimulation in the home environment, finding positive associations between maternal education and home stimulation that were associated with enhanced cognitive development. Such evidence has spurred the interest in and development of ways to assess specific variables within the context of the home (e.g., mother-child interactions; Rasheed & Yousafzai, 2015). Given the significance of the home environment on early childhood development, the home environment is sometimes used as a proxy for early childhood care and education (Iltus, 2006). Increased parent responsiveness has been linked with cognitive development and later learning (Bradley et al., 2001). Further, Evans et al. (2010) explained how maternal responsiveness mediates the effect of home crowding on cognitive development.

#### 1.1.2 Physical environmental characteristics (cleanliness, safety, healthfulness)

Physical factors in the environment have also been shown to affect child development (Ferguson, Cassells, MacAllister, & Evans, 2013). Shaw (2004) reviewed the link between physical health and the environment, stating that children in poorer quality housing are more likely to experience increased rates of infection. Research has pointed to higher rates of physical illness such as skin infections among homes without working toilet facilities (Bailie et al., 2005). Such infectious diseases can have both direct and indirect effects on child physical and psychological development (Walker et al., 2007). Additionally, poor environmental safety may lead to increased risk of exposure to neurotoxins, such as lead, which can lead to cognitive impairments (Kippler et al., 2012, Lanphear et al., 2005, Needleman et al., 1979). Home crowding has been identified by several researchers as having one of the strongest associations with scores on the HOME (Bradley and Caldwell, 1984, Johnson et al., 1984), with research demonstrating that home crowding may lead to social withdrawal and reduced parental responsivity (e.g., Bradley and Caldwell, 1984, Evans, 2006). Given that both physical and interpersonal/experiential factors influence developmental outcomes, it is important for a measure of home environment to account for both parental responsivity to children and the physical characteristics of the environment.

### 1.2 Assessment of home environment

#### 1.2.1 Methods for assessing environmental characteristics

Several tools may be used for gathering information about and assessing the home environment, including observations, interviews, and rating scales. Observational methods (such as the HOME; Caldwell and Bradley, 1984, Caldwell and Bradley, 2003) rely on trained observers to assess and evaluate the home environment, the behavior of the child, and the interactions between a child and an adult. Interview methods use information collected through parents'/caregivers' responses to a series of questions asked by an interviewer about the environmental characteristics of the home and the behavior of the child. Leventhal, Selner-O'Hagen, Brooks-Gunn, Bingenheimer, and Earls (2004) developed an interview tool that was based on the HOME to assess home environments in Chicago neighborhoods, also requiring a trained professional in the administration of the interview. Rating scales are completed by informants who know the child well, such as parents or caregivers, and do not require much time or expertise for scoring and interpretation. For instance, following the development of the HOME, Frankenburg and Coons (1986) developed a similar measurement tool to evaluate the quality of one's home environment. However, rather than relying on the direct observation of the environment by a trained professional, the questionnaire was completed by the parents and/or caregivers. Inherent limitations are relevant with both interviews and self-report rating scales in the form of response bias. For example, social desirability may influence parent responses (Paulhus, 2002). Additional concerns included extreme response styles and acquiescent responding (Jackson and Messick, 1958, Krosnick, 1999). Thus, direct observation of an environment has the potential to improve objectivity through the use of trained raters. Regardless of the type of assessment tool used (rating scale, interview, or observation), the scores produced by the measure must be reliable and valid among members of the population with which they are used (AERA, APA, & NCME, 2014).

#### 1.2.2 Home observation for measurement of the environment

The Home Observation for Measurement of the Environment (HOME; Caldwell and Bradley, 1984, Caldwell and Bradley, 2003), is the most widely recognized and commonly used instrument for evaluating the quality of the home environment and has been frequently revised and adapted. Originally developed from the work of Elardo, Bradley, and Caldwell (1975), the HOME is an observational tool that allows trained observers to quantify observations related to mother-child interactions, family living patterns and habits, orderliness of the physical home environment, and the potential of the observed environment to positively influence development. It was developed to evaluate aspects of the environment that are believed to be important for development, such as parental responsivity and opportunities to learn (Elardo & Bradley, 1981).

The HOME has been used for a number of purposes, including evaluating interventions (Brotman et al., 2005) and assessing the effect of parenting on a number of outcome variables, including: academic achievement (Bradley & Caldwell, 1984), executive functioning Blair, Raver, and Berry (2014)), language development (Elardo, Bradley, & Caldwell, 1977), and prenatal/perinatal exposure to neurotoxins (Walkowiak et al., 2001). Brooks-Gunn, Klebanov, and Liaw (1995) demonstrated the use of the HOME to measure risk-factors and outcomes associated with families living in poverty. The relationship between the HOME and cognitive development has also been well documented over time Elardo et al. (1975); Bradley and Caldwell, 1976, Bradley et al., 1988, Luster and Dubow, 1992). Parental attachment has also been evaluated utilizing the HOME (NICHD Early Child Care Research Network, 2001). Within the United States, attempts have been made to evaluate the measurement equivalence/invariance of the HOME (Bradley et al., 1996, Whiteside-Mansell et al., 2003). Bingenheimer, Raudenbush, Leventhal, and Brooks-Gunn (2005) evaluated the HOME for its equivalence across racial and ethnic groups. Consistent with the review completed by Bradley et al. (1996), while measurement equivalence was demonstrated between Black and White families on the HOME item functioning, the scale did not function similarly among Hispanic families. Thus, while the psychometric properties of the HOME with sample populations consistent with the original groups studied using the HOME have demonstrated equivalence (e.g., Saudino and Plomin, 1997, Shaw and Vondra, 1995, Prodromidis et al., 1995), research with other racial and ethnic groups has been mixed (Totsika & Sylva, 2004).

#### 1.2.3 Cross-cultural adaptations of the HOME

The HOME has been shown to predict a number of child developmental outcomes within the United States (Totsika & Sylva, 2004). In order to use the inventory outside of the US, however, adaptations have been necessary (Black et al., 2004, Bradley et al., 1996). A review of the cross-cultural uses of the HOME scale by Bradley et al. (1996) revealed important differences among developing nations, relative to history, sociocultural norms, and politics, highlighting the need for the equivalence of the HOME to be demonstrated across cultures before broad generalizations are made. Rather, the authors highlight the limitations of the HOME when administered without adaptations, as key home factors likely differ significantly between cultures. Generally, items associated with cognitively stimulating environments appeared to demonstrate the highest degree of equivalence across cultures. However, equivalence of the HOME factors must be weighed against the relevance in different cultures. Several researchers have highlighted the need for and proposed guidelines for the development of cross-cultural assessment tools (Hambleton, 2001, Peña, 2007, Van Widenfelt et al., 2005). Geisinger (1994) outlines several steps in adapting the use of normative assessments for cross-cultural use, particularly the importance of ensuring that the same psychological construct is being measured across groups.

More recent uses of the HOME have also included participants from Thailand (Williams et al., 2003) and Bangladesh (Black et al., 2004). As noted by Bradley et al. (1996), researchers have examined the factor structure of the HOME in several different countries, and the findings have differed widely ranging from identification of a three-factor structure in Costa Rica (Stevens & Bakeman, 1985) to a seven-factor one (with many factors having very poor reliability, e.g., < 0.50) in a study of Hispanic-Americans (Bradley et al., 1994a, Bradley et al., 1994b). Notably, both the study locations and the factor analytic techniques differed across research studies, so it is unclear whether the discrepant findings are the result of cultural differences or methodological ones. In summary, due to inconsistency in methods and findings across cultural groups, the factor structure of the original HOME is unknown. Moreover, the factor structure of the Bangladeshi adaptation of the HOME that was used in this study, to our knowledge has never been examined. The current paper adds to the existing knowledge base by examining the measurement equivalence/invariance of the HOME across international sites.

#### 1.2.4 Measurement equivalence/invariance

Measurement equivalence/invariance refers to the extent to which an assessment tool measures the intended construct in a similar way across groups (Drasgow, 1984, Pendergast et al., 2017). Moreover, Pendergast and colleagues identified a number of instances when ME/I is important to understanding how tests and measures are used across different populations, such as evaluating test bias or differences in item functioning across cultural groups. To our knowledge, no study has ever explicitly tested the ME/I of HOME scores across international groups. According to Vandenberg and Lance (2000), “The establishment of measurement equivalence/invariance across groups is a necessary and logical prerequisite to conducting substantive cross-group comparisons (e.g., tests of group mean differences, invariance of structural parameter estimates)” (p. 1). In other words, researchers need to provide evidence that scores on a measurement tool are equivalent/invariant across groups before mean scores across groups can be compared. Measurement equivalence/invariance is important when examining the psychometric properties of a particular measure across groups, such as gender (Marsh, 1985, Marsh, 1987), age (Marsh & Hocevar, 1985) or cultural groups (Riordan & Vandenberg, 1994).

### 1.3 Present study

The purpose of the current study was to identify subsets of HOME items that yielded scores with evidence of validity and measurement equivalence/invariance across the eight international MAL-ED sites. Notably, measurement equivalence/invariance (ME/I) is considered to be a necessary prerequisite for comparing mean scores across groups (Vandenberg and Lance, 2000, Pendergast et al., 2017). Moreover, ME/I is one piece of evidence that allows for increased confidence in testing primary hypotheses related to home environment across groups.

## 2 Methods

### 2.1 Overview of MAL-ED study

The MAL-ED study (The MAL-ED Network Investigators, 2014) is a multi-national and multi-disciplinary, observational, prospective, cohort study conducted at eight international sites. The primary objective of MAL-ED is to examine the extent to which nutrition, enteric infections, and gut inflammation and permeability influence growth and cognitive and psychological development of children in resource-poor environments. Many factors influencing child development were measured, including economic resources, maternal education, reasoning and depression as well as aspects of the home environment.

### 2.2 Participants

The MAL-ED sample is comprised of mothers and infants from research sites located in eight different nations: Dhaka, Bangladesh (BGD; *n* = 237); Vellore, India (INV; *n* = 235); Bhaktapur, Nepal (NEB; *n* = 235); Naushahro Feroze, Pakistan (PKN; *n* = 246); Fortaleza, Brazil (BRF; *n* = 198); Loreto, Peru (PEL; *n* = 257); Venda, South Africa (SAV; *n* = 231); Haydom, Tanzania (TZH; *n* = 240). Community-based sampling was used in this study whereby all families who met criteria (e.g., singleton infant, weight above 1500 g) within a selected geographic region were invited to participate. More detailed descriptions of the demographic characteristics of each site are provided in Appendix A. A more thorough description of the sites, recruitment procedures, adaptations and translation are described elsewhere (The MAL-ED Network Investigators, 2014). Of the 2145 infants enrolled within 17 days of birth, 1598 infants (74%) were followed to at least 24 months of age, when the HOME was administered. The targeted window for administration of the HOME was 24 months of age (± 15 days).

### 2.3 Measure

#### 2.3.1 Home Observation for the Measurement of the Environment (HOME)

The HOME has four versions: Infant/Toddler (0–2 years), Early Childhood (3–6 years), Middle Childhood (6–10), and Early Adolescence (10–15 years). The version of the HOME scale used in the MAL-ED cohort study (provided in Appendix B) was originally adapted by Black et al. (2004) for use among a population of infants in Bangladesh. These modifications, made in conjunction with anthropologists, ensured that the HOME items were culturally appropriate to Bangladeshi communities. Black et al.'s adapted version used in MAL-ED is comprised of 48 items on six subscales: parent responsiveness, avoidance of restriction and punishment, organization of the environment, appropriate play materials, parental involvement, and variety in daily stimulation. The purpose of the adaptation was to ensure that items were culturally appropriate to the community in which it was being utilized. Further, additional items were added that addressed the physical layout of the environment. However, the psychometric validity of this adapted version of the HOME has not yet been examined outside of Bangladesh. Establishing psychometric support for the use of this measure in further analyses associated with the MAL-ED study is necessary, and the current research sought to address that gap.

### 2.4 Procedure

#### 2.4.1 Overview

For a detailed review of the methods and procedures for the MAL-ED study see Murray-Kolb et al. (2014) and The MAL-ED Network Investigators (2014). A review of procedures specific to the HOME Inventory is provided here. Prior to beginning our research, the HOME Inventory was translated into each target language and back translated by an individual who had never seen the original form. The translations were compared by individuals with relevant measurement and cultural expertise and adaptations were made as needed. Subsequently, the HOME Inventory was administered at 24 months of age to assess the characteristics of the home environment. To ensure uniformity of administration and observation, regional on-site trainings were conducted at the eight field sites. Each training session at each of the eight field sites lasted 5–6 days with 1 day devoted to training in the adapted HOME inventory. Training included both didactic components and practice administrations.

#### 2.4.2 Personnel selection

The HOME was administered by psychological research assistants (PRAs) at each site. The criteria agreed upon for the selection of a PRA included a level of education that is comparable to a Master's degree in psychology or child development and fluency in speaking the local language. Six of the MAL-ED sites found PRAs with the required criteria. Two of the sites had difficulty identifying individuals with these qualifications and, therefore, the PRAs in these two sites were selected based on their education level (minimum education of 14 years in Pakistan and 11 years in Tanzania), knowledge of child development, and previous experience working on research studies. PRAs who administered the HOME underwent training which included supervised practice administrations.

#### 2.4.3 HOME administration

The HOME assessment was scheduled at a time when the primary caregiver and child were at home and the child was awake. The PRAs visited and worked with the families regularly and, in most instances, were known to the participants. Prior to beginning the HOME, the PRAs engaged in conversation and rapport building with the families. Subsequently, they informed the families that they would need to observe the child and the home setting for approximately 1 h. The HOME form was completed over the course of the hour, and any unanswered questions were posed to the child's primary caregiver after the observation was complete. Each item had guidelines for scoring and all items on the forms received binary scores (+) or (−). PRAs scored the items based on their observations during the visit and, when appropriate asked the caregiver to provide information. All responses were recorded on paper-and-pencil forms.

#### 2.4.4 Quality assurance

As the assessment of the HOME was carried out in eight, culturally diverse sites, many quality assurance procedures were implemented to ensure consistency in assessment and scoring methods across sites. These procedures included training, weekly conference calls with key team members, random reviews of materials and forms, and assessments of inter-rater reliability conducted by senior psychologists. Weekly calls ensured that a discussion forum was available so that all sites had the opportunity to discuss relevant issues and barriers, had common understanding of expectations, and that any change was communicated to each site. Ten percent of forms were checked by the senior psychologist at each site by random selection. Inter-rater reliability, as indicated in Murray-Kolb et al. (2014) of the HOME was addressed through simultaneous scoring by the PRA and the senior psychologist. Supervisors and PRAs were required to have 85% correspondence in ratings before PRAs could administer the scale independently (and in order to continue independent administration). All variations between the PRA and senior psychologist were discussed between the two raters.

### 2.5 Data analysis

#### 2.5.1 Overview

To accomplish our objective of deriving a measure of home environmental characteristics that is likely to be useful across diverse groups in many settings, a five-pronged approach was used to examine the validity of the scores from each scale across the eight groups. Prior to analyses, the sample was divided into two randomly selected subsamples – one for exploratory factor analysis (EFA; *n* = 940) and one for confirmatory factor analysis (CFA; *n* = 939). In the first step, EFA was conducted to better understand the nature of the factor structure in this sample. In the second step, the internal consistency of scores was examined with the EFA subsample (all eight sites). For the third step, CFAs were conducted based on the CFA subsample (*n* = 939) with data from all sites. Next, a MIMIC modeling approach was employed (see Jones, 2006, Woods et al., 2009; and Pendergast et al., 2014 for reviews and illustrations of the MIMIC modeling approach to evaluating ME/I). Finally, convergent validity of HOME scores and crowding was examined. Details of the procedures used in each analysis are provided below.

#### 2.5.2 Exploratory factor analysis and internal consistency

Common factor analysis (principal axis factoring, promax rotation) was selected because it is the preferred technique for identification of latent factor structures (Briggs and MacCallum, 2003, Fabrigar et al., 1999). Exploratory factor analyses were conducted in SPSS (version 22) based on tetrachoric matrices (Lorenzo-Seva & Ferrando, 2012). Three methods were employed to determine the number of factors to retain: parallel analysis (PA; Horn, 1965, Watkins, 2006), minimum average partials (MAP; Velicer, 1976), and a visual scree test (Cattell, 1966, Henson and Roberts, 2006). Pattern coefficients ≥ 0.30 on only one factor were considered salient (Stevens, 2002). Factor structures with (a) ≥ three items with salient pattern coefficients; (b) evidence of reliability (e.g., internal consistency ≥ 0.70 or support based on item response theory analyses); and (c) a conceptually or theoretically meaningful pattern were considered to be adequate and were submitted for confirmatory factor analysis.

#### 2.5.3 Baseline confirmatory factor analysis

CFAs were conducted in MPlus 6.12 using mean and variance adjusted weighted least squares estimation (WLSMV). Goodness of fit was evaluated based on multiple criteria (Tanaka, 1993): (a) root mean square error of approximation (RMSEA) values < 0.075, (b) standardized root mean square residual (SRMR) values < 0.08, and (c) comparative fit index (CFI) values > 0.90 (Hu and Bentler, 1995, Markland, 2007). Although more stringent criteria exist (e.g., CFI > 0.95; Hu & Bentler, 1999), rigid adherence to these more stringent criteria could lead to inappropriate rejection of well-fitting models (e.g., Marsh, Hau, & Wen, 2004). Therefore, relatively liberal criteria (e.g., CFI > 0.90) were utilized, and multiple fit indices were examined (Markland, 2007).

#### 2.5.4 MIMIC modeling analyses

When conducting cross-cultural research, it is important for researchers to evaluate whether their measures operate similarly across groups. Two important principles related to measurement in cross-cultural research are measurement equivalence/invariance (ME/I; see Pendergast et al., 2017 for a review) and differential item functioning (DIF; see Zumbo, 2007, for a detailed discussion of ME/I and DIF). The two most commonly used techniques for examining ME/I and DIF are multi-group confirmatory analysis and item response theory analysis. However, both of those approaches are typically used in analyses examining a small number of groups and may not be appropriate for analyses across many groups (e.g., Asparouhov and Muthén, 2014, Pendergast et al., 2017).

Multiple indicators multiple causes (MIMIC; Jöreskog & Goldberger, 1975) modeling is similar to CFA but allows for examination of DIF through the inclusion of covariates to test the influence of extraneous variables (e.g., nationality, gender, race, etc.) on the factor structure. This allows the researcher to evaluate the equivalence of scores from a measure across groups (Muthén, 1989, Jones, 2006). Advantages to MIMIC modeling, as noted by Woods et al. (2009), include ease in modeling multidimensional constructs (such as home environment), availability of high quality software, capacity for modeling categorical data (as is used in the HOME), and ability to examine DIF across multiple groups simultaneously. To examine differential item functioning (DIF) across the eight international sites, a MIMIC modeling approach was used in this study.

The MIMIC modeling analyses began by following the procedures recommended by Shih and Wang (2009). First, a subset of one or more DIF-free items were identified and specified as anchor items in subsequent analyses (Shih and Wang, 2009, Woods et al., 2009). Subsequently, the stepwise method for DIF analyses described by Jones (2006) was used, and, accordingly, multiple interim MIMIC models were estimated. Next, the remaining (non-anchored) items were tested for DIF using an iterative process whereby the items were tested one at a time, and modification indices were inspected after each analysis. If the modification indices indicated that freeing a path between an item and a cultural (site) variable would meaningfully influence the χ^2^ value, then the item was considered to have DIF. Finally, effect sizes were calculated using MLR estimation to derive proportional odds ratio (OR) estimates and interpreted according to the Cole, Kawachi, Maller, and Berkman (2000) guidelines.

#### 2.5.5 Convergent validity

Convergent validity analyses included a measure of home crowding. Home crowding was defined in the larger MAL-ED study as the number of persons living in the home divided by the number of rooms in the home. Correlations between each factor obtained from the factor analyses of the HOME were obtained. Pearson correlation coefficients were then computed for each obtained factor and crowding. Moderate relationships, whereby increased crowding predicting poorer home environmental conditions were predicted based on prior literature (Bradley and Putnick, 2012, Conley, 2001, Matheny et al., 1995).

## 3 Results

### 3.1 Preliminary analyses

The version of the HOME administered in this study included 48 items. Before beginning primary analyses, all five items from the original Avoidance of Restriction and Punishment subscale were removed from the dataset. This subscale included items that inquired about caregiver behaviors such as spanking or slapping the child, shouting at the child, and expressing overt annoyance toward the child. Such behaviors were quite rare in our sample (e.g., endorsed by 0–2% of respondents at many sites) – creating numerous empty cells and thereby violating the assumptions of categorical factor analysis.

### 3.2 Exploratory factor analyses

The remaining 43 items from the HOME were submitted for EFA. Bartlett's test of sphericity was statistically significant (*p* < 0.001). The Kaiser-Meyer-Olkin statistic was 0.81 lending support for the suitability of the correlation matrix for factor analysis (Kaiser, 1974). The recommended number of factors varied widely. Parallel analysis suggested 6 factors, visual inspection of the scree plot indicated 7 factors, and minimum average partials suggested 16 factors. All of these factor solutions were attempted but rejected because (a) one or more factors had an inadequate number of items (fewer than 3) with salient pattern coefficients (> 0.30), or (b) one or more factors with insufficient reliability (< 0.70). Subsequently, four- and five-factor solutions were attempted and rejected for the same reasons. The three-factor solution met the aforementioned criteria and was retained. Items that did not load on any factor were removed from analyses, and the analyses were conducted without them.

The final three-factor solution met all a priori criteria. It was comprised of factors that appeared to reflect the following dimensions: Emotional and Verbal Responsivity; Clean and Safe Environment, and Child Cleanliness. Pattern coefficients and alpha values for all factors are reported in Table 1. The Emotional and Verbal Responsivity factor was comprised of 13 items that appeared to reflect the extent to which the primary caregiver (usually the mother) responded to the child's emotional states and attempts at verbalization. Examples of items on this factor include, Caregiver spontaneously praises child's qualities or behavior and Caregiver spontaneously vocalizes to the child.” Five items were included on the Clean and Safe Environment factor. This factor appeared to reflect the extent to which features of the environment were conducive to the safety and health of the child, reflecting more permanent features of the child's environment. Examples of items on this factor include “The house is relatively light” and “The stove is located in a relatively safe area.” The third factor, Child Cleanliness, included four items that reflected the cleanliness of the child's hair, clothes, etc. Examples of items on this factor include “The child is relatively clean, with no offensive odor” and “The child's hair is relatively clean.” Notably, one item, “House is neat and orderly,” cross-loaded on both the Clean and Safe Environment and the Child Cleanliness factor. In factor analysis, cross-loaded items are often problematic. Cross-loaded items indicate shared contributions to multiple factors, raising questions about the independence of extracted factors with regard to the common indicator. Therefore, this item was tested as loading on both factors, separately, in CFA, and model fit was compared.

**Table 1:** Pattern matrix for exploratory factor analysis (principal axis factoring) with promax (Oblique) Rotation of the Home Observation for the Measurement of the Environment (HOME).

Items (abbreviated)	Emotional and verbal responsivity	Child cleanliness	Environmental safety and healthfulness
Spontaneous praise for child 2 ×	**.693**	.035	−.059
Respond to vocalization by kid	**.603**	.032	−.023
Initiates verbal exchange with observer	**.588**	−.146	.063
Spontaneous vocalize to kid	**.580**	−.076	.016
Positive response	**.568**	−.041	−.070
Tells name of object	**.566**	−.135	.015
Affectionate with child	**.509**	.151	.129
Talks to child often	**.495**	.053	−.141
Smiles at child	**.464**	.055	.000
Encourages development	**.430**	.225	−.020
Believe can modify behavior	**.429**	.040	−.087
Expresses ideas freely	**.386**	−.043	.142
Positive feeling conveyed	**.372**	.016	.058
Child clean	−.028	**.905**	−.079
Clothes relatively clean	.008	**.831**	.078
Hair neat/washed recently	.063	**.807**	.004
Stove in safe area	.050	−.041	**.811**
Play area relatively safe free of hazards	.061	−.036	**.782**
House relatively light	−.089	−.012	**.517**
House neat orderly	−.103	**.341**	**.366**
Taken to health clinic regularly	−.031	.158	**.317**
Alpha coefficient	.815	.724	.811
		.704^a^	.890^a^

*Note*. Factor loadings > .30 are in boldface. Alpha estimates were based on EFA subsample.

a Alpha value without “Neat and orderly” item (cross loaded item).

### 3.3 Confirmatory factor analyses

The three-factor model of HOME scores was examined in CFA based on an independent subsample (*n* = 939). The model was determined to be a good fit to the data based on several criteria (e.g., CFI = 0.94; RMSEA = 0.055 (CI = 0.051–0.060)). All factors were allowed to correlate (inter-factor correlations ranged from − 0.04 to 0.68). No post-hoc modifications were used. However, two models (specified a priori) were run and compared: one with the item “House is neat and orderly” loading on the Clean and Safe Environment factor and one with that item loading on the Child Cleanliness factor. The model where the item loaded on the Child Cleanliness factor had superior fit (alternative model CFI = 0.89; RMSEA = 0.08) and was retained.

### 3.4 MIMIC modeling analyses

#### 3.4.1 Overview of DIF analyses

MIMIC analyses were conducted based on the entire sample. After identification of anchor items, DIF analyses proceeded in a stepwise fashion as described by Jones (2006) involving estimation of multiple interim MIMIC models (see Pendergast et al., 2014, for a more detailed illustration of this process). Subsequently, a final model was tested allowing DIF where indicated (based on findings from the interim models). A listing of items and sites for which DIF was allowed can be found in Table 2. The final model, which allowed for DIF, demonstrated good fit to the data (e.g., CFI = 0.96; RMSEA = 0.05). The final MIMIC model was re-estimated using the maximum likelihood estimator with robust standard errors (MLR) to examine the effect sizes associated with DIF by calculating proportional odds ratio (OR) estimates. As suggested by Cole et al. (2000), OR values > 2.0 or < 0.5 were considered to be indicative of meaningful DIF. Parameter estimates and OR estimates are reported in Table 2.

**Table 2:** Parameter estimates and differential item functioning of HOME items across seven sites.

Items	BGD	BRF	INV	NPB	PEL	PKN	SAV	TZH
	PE (OR)	PE(OR)	PE(OR)	PE(OR)	PE(OR)	PE(OR)	PE(OR)	PE(OR)
Caregiver vocalizes to child	−.39(.86)	**.17(4.51)**	NT	**−.13(.16)**	–	–	–	NT
Caregiver responds to child	.20(1.27)	**.11(3.70)**	NT	NT	–	**−.09(.30)**	–	NT
Caregiver teaches names of objects^a^	NT	–	NT	–	–	–	–	NT
Caregiver initiates conversation	.**24(.06)**	**.16(3.91)**	NT	**.34(13.42)**	–	–	–	NT
Caregiver expresses ideas freely	–	–	NT	–		**−.33(.09)**	–	NT
Caregiver praises child	**.44(9.92)**	–	NT	–	**.55 (3.43)**	–	–	NT
Caregiver discusses child positively	.68(.70)	–	NT	–	–	**−.15(.23)**	–	NT
Caregiver is affectionate	**.78(384.01)**	**.12(2.41)**	NT	**.28(6.62)**	**−.66 (.39)**	**−.43(0.05)**	–	NT
Caregiver responds positively to child	0.11(.53)	**.15(4.05)**	NT	**0.25(5.40)**	.27 (1.90)	**.15(2.15)**	**.19(5.74)**	NT
Caregiver smiles at child	NT	**.21(6.10)**	NT	**−.20(.11)**	.**44(2.92)**	–	**.18(4.72)**	NT
Caregiver talks to child while working	**.70(116.26)**	**.17(3.30)**	NT	**−.20(.18)**	–	–	–	NT
Caregiver encourages development	**.62(55.81)**	**.13(2.44)**	NT	**.21(3.66)**	–	**−.22(22)**	**.13(2.82)**	NT
CG believes behavior is modifiable	**.70(118.81)**	–	NT	**.22(3.93)**		**−.13(.44)**	**.20(4.46)**	NT
Factor I total items with meaningful DIF	6	8	NT	8	3	7	4	NT
Child regularly visits health clinic	**.49(67.61)**	**.19(4.23)**	–	−.10(.58)	–	–	**−.33(.13)**	**−.39(.36)**
Play area is relatively safe^a^	**−.16(.18)**	–	–	–	–	–	–	–
Stove is in a safe area	–	–	–	–	–	**−.18(.28)**		
House is relatively light	–	–	–	–	–	–	–	**−.20(.23)**
Factor II total items with meaningful DIF	2	1	0	0	0	1	1	2
House is neat and orderly	NT	–	–	**−.21(.19)**		**−.21(.18)**	.19(5.81)	**−.89(.24)**
Child is relatively clean	NT	–	–	–	–	**−.12(.15)**		–
Child's hair is relatively clean^a^	NT	–	.14(4.75)	–	–	–	–	–
Child's clothes are clean	NT	–	–	**−.07(.19)**	**.41**	–	−.07(24)	**−.10(0.09)**
Factor III total items with meaningful DIF	NT	0	1	2	1	2	2	2

*Note*. – indicates that there was no DIF and the parameters were not estimated. PE = Standardized Parameter Estimate; Odds ratios are reported in parentheses, and items deemed to have meaningful DIF (ORs > 2.0 or <.50) are bolded. NT refers to items that were not tested for DIF due to empty cells (an absence of participants who endorsed or failed to endorse the item.) Study sites were not comprised of samples that were demographically representative of the countries in which they were located and should not be interpreted as such.

a Denotes items used as anchor items in analyses.

#### 3.4.2 Emotional and verbal responsivity DIF

For factor I, Emotional and Verbal Responsivity (EVR), there were statistically significant paths between the EVR factor and five sites. Four sites scored significantly lower in regard to overall EVR scores: (BGD: PE^2^ = − 0.39, *p* < 0.001; BRF: PE = − 0.34, *p < 0*.001; PEL: PE = − 0.17, *p* = 0.001; and PKN: PE = − 0.10, *p* = 0.016). One site scored significantly higher on the overall EVR score (NPB: PE = 0.28, *p* < 0.001), and there was no statistically significant difference for the South Africa site. Notably, invariance could not be tested for two sites, INV and TZH, due to extremely limited variance by which observers endorsed items for every participant or nearly every participant which resulted in numerous empty cells. At the item level, after controlling for differences on the overall factor, a high number of items on each factor displayed statistically significant and meaningful DIF as shown in Table 2.

#### 3.4.3 Clean and safe environment DIF

On factor II, Clean and Safe Environment (CSE), statistically significant paths occurred between the CSE factor and six sites. Four sites scored significantly higher in regard to the overall CSE scores (INV: PE = 0.52, *p* < 0.001; NPB: PE = 0.12, *p* = 0.001; PEL: PE = 0.33, *p* < 0.001; SAV: PE = 0.56, *p* < 0.001). Two sites scored significantly lower: (PKN: PE = − 0.38, *p* < 0.001; TZH: PE = − 0.54, *p* < 0.001), and no statistically significant differences were evident for the BGD and BRF sites. At the item level, after controlling for differences on the overall factor, DIF was minimal. Three sites had zero items with meaningful DIF, three sites had only one item with meaningful DIF, and two sites had two DIF items (reported in Table 2). Thus, the CSE factor was largely comparable across sites.

#### 3.4.4 Child cleanliness DIF

For factor III, Child Cleanliness (CC), statistically significant paths emerged between the CC factor and overall scores from seven sites. Four sites scored significantly higher in regard to the overall CC score (BRF: PE = 0.15, *p* = 0.004; NPB: PE = 0.36, *p* < 0.001; PEL: PE = 0.10, *p* = 0.025; and SAV: PE = 0.32, *p* < 0.001). Three sites scored significantly lower in regard to the overall CC score (INV: PE = − 0.26, *p* < 0.001; PKN: PE = − 0.21, *p* < 0.001; and TZH: PE = − 0.21, *p* < 0.001). One site, BGD, could not be tested for DIF on the CC factor due to extremely limited variability in responses to items. At the item level, after controlling for the overall factor scores, evidence of a moderate degree of DIF was present. One site had zero items with meaningful DIF, two sites had one item, and four sites had two items (reported in Table 2). Thus, the CC factor was somewhat comparable across sites.

### 3.5 Convergent validity

Pearson's correlations were analyzed based on unit-weighted factor scores, first between each of the three factors obtained through EFA (see Table 3). Emotional and verbal responsivity was significantly correlated with environmental safety (*r* = 0.270, *p* < 0.001), although, not surprisingly, not with cleanliness (*r* = 0.057, *p* = 0.051). Environmental safety was positively correlated with cleanliness (*r* = 0.387, *p* < 0.001).

**Table 3:** Summary of Pearson correlation coefficients, means, and standard deviations for scores on the EVR, CSE, CC, and crowding.

Measure	1	2	3	4
1. EVR	–			
2. CSE	.27⁎⁎	–		
3. CC	.06	.39⁎⁎	–	
4. Crowding	−.03	−.39⁎⁎	−.34⁎⁎	–
M	10.71	3.20	3.26	2.65
SD	2.65	1.12	1.21	1.67

*Note*. EVR = Emotional and Verbal Responsivity; CSE = Clean and Safe Environment; CC = Child Cleanliness.

⁎⁎ *p* < 0.01 based on combined EFA and CFA subsamples.

The association between each of three HOME factors with environmental crowding (number of individuals living in the home divided by number of rooms used for sleeping) was also examined. Environmental crowding was not associated with emotional and verbal responsivity (*r* = − 0.028, *p* = 0.338). However, as expected based on prior research (Bradley and Caldwell, 1984, Johnson et al., 1984), crowding had low to moderate correlations with both environmental safety (*r* = − 0.385, *p* < 0.001) and cleanliness (*r* = − 0.336, *p* < 0.001), both associations reaching statistical significance.

## 4 Discussion

Bronfenbrenner (1977), in his ecological systems theory, suggested that child development must be interpreted in the context of the child's environment. The empirical literature clearly demonstrates that early childhood environment has a meaningful and lasting impact on child development (Andrade et al., 2005, Bradley and Corwyn, 2002, Leventhal and Brooks-Gunn, 2000, Pendergast and Kaplan, 2015, Shaw, 2004, Shonkoff et al., 2012). This research examines the validity of scores from an observational measure of the home environment, the primary context in which child development occurs. Examining the role that environmental factors play in child development across cultures is challenging for researchers because environmental contexts vary widely across cultural groups. Moreover, the extent to which many measures of early childhood environment are reliable and valid across multiple cultural groups is unknown. This study examined the validity of the HOME across eight international sites that differ widely in regard to culture, language, and economic resources.

### 4.1 Overview of findings

The findings from this study largely support the psychometric properties of scores from the HOME as measures of Emotional and Verbal Responsivity, Clean and Safe Environment, and Child Cleanliness in the eight low- and middle-income settings examined in this study. The validity scores of some of the other original subscales (e.g., avoidance of physical punishment; variety in stimulation) were not supported with this sample. Moreover, the extent to which each subscale was supported, as well as the degree of measurement equivalence across sites, varies by subscale and site.

#### 4.1.1 Emotional and verbal responsivity

Specifically, these findings indicate that HOME scores measure Emotional and Verbal Responsivity fairly well (in regard to structural validity) for six of our eight research sites located in six countries across three continents BGD, BRF, NPB, PKN, PEL, and SAV, while not functioning well at the Indian and Tanzanian MAL-ED sites. This suggests that some aspects of maternal emotional and verbal responsivity can be assessed in diverse groups, while in some areas the scores are less likely to be useful in research or clinical contexts. Nonetheless, support for the assessment of emotional and verbal responsivity across cultures is a critical finding from this study and can be used to inform future research, as this construct is closely related to child development. The dyadic interactions between parent/caregiver and child are important for the development of cognitive skills and language from an early age (e.g., Sameroff & Fiese, 2000). Thus, further research in this area should focus on determination of the impact of emotional and verbal responsivity on cognitive development across cultures.

#### 4.1.2 Clean and safe environment

Clean and safe environments for children are critical to ensuring positive developmental trajectories. Increased rates of infection have been linked to the physical environment (Shaw, 2004, Bailie et al., 2005). The current findings support the use of the HOME Clean and Safe Environment subscale across the sites examined in this study and suggest that it is likely to be an effective measure for future researchers examining diverse populations in low- and middle-income nations. A high degree of measurement equivalence was demonstrated across sites – as 50–100% of items displayed no meaningful DIF in each site. In addition, positive correlations were observed between home crowding and environmental safety.

Children living in crowded conditions are at a higher risk for injury, including burns (Delgado et al., 2002) and respiratory infection (Cardoso et al., 2004). Given the relationship of crowding with both the risk of injury and the spread of infectious disease, identification of proper safety measures taken within the home (e.g., the placement of the stove within the home) and steps to prevent the spread of infectious diseases (e.g., access to healthcare), findings of convergent validity of our home safety and crowding variables are important and support the use of the scale.

#### 4.1.3 Child cleanliness

These findings suggest that the HOME Child Cleanliness factor effectively measured Child Cleanliness in seven of the eight MAL-ED sites located on three continents (i.e., all sites except Bangladesh). Moreover, a high degree of measurement equivalence was evidenced across sites for this subscale as 50–100% of items displayed no meaningful DIF in each of the seven sites. This factor was also correlated positively with home crowding. These findings support the use of the HOME Child Cleanliness subscale across the seven indicated sites and suggest that it is a promising tool for future researchers examining diverse populations in low- and middle-income nations.

Environmental factors that contribute to the health and safety of the child are also consistently illustrated in empirical literature (e.g., Ferguson et al., 2013, Grandjean and Landrigan, 2006, Lanphear et al., 2005, Needleman et al., 1979, Walkowiak et al., 2001). Personal hygiene, nutrition and the risk of infection are also linked to cognitive and psychological development (Aiello and Larson, 2002, Walker et al., 2007). Thus, a measure of a child's cleanliness can serve as an indicator of the hygiene practices of the family that may contribute to the spread of infectious diseases. The relationship between infectious disease and cognitive development, for instance, is the subject of ongoing research with some evidence suggesting that chronic infections may contribute to adverse effects on cognitive development and academic achievement (Guerrant et al., 2013, Guerrant et al., 2008, Walker et al., 2007). As a physical characteristic of the home, the relationship between home crowding and child cleanliness is also relevant, given the possibility of increased transmission of infectious disease among members of a household (e.g., Jaine, Baker, & Venugopal, 2011). Thus, attention to child cleanliness is critical for reducing levels of infectious disease transmission in crowded homes.

### 4.2 Implications

The current research sought to evaluate the psychometric properties of the HOME across cultural groups. Culturally relevant modifications to the HOME have been previously made in evaluating home environment variables (e.g., Black et al., 2004). To our knowledge, however, a measurement equivalence/invariance evaluation of the HOME scale has never been conducted outside the US, despite suggestion by the original author encouraging the use of the HOME across cultural groups (Bradley et al., 1996). Thus, although the influence of home environment on various cognitive skills and developmental trajectories appears to remain constant across cultures, the specific indicators of the home environment that are relevant to individual cultural groups is more variable. As the sample population from which the MAL-ED data is drawn was not selected to be representative of entire regional populations, the indicators and factors that emerged as relevant for the particular cultural groups specific to these analyses may not generalize to entire regional populations.

### 4.3 Strengths, limitations, and future directions

Strengths of the current study include the use of a large international sample from diverse communities across eight low- and middle-income countries. The HOME measure, although previously used with international groups (Black et al., 2004, Bradley et al., 1996), has not been subject to an analysis of measurement equivalence/invariance cross culturally. Thus, the current study contributes to the existing research base in a unique way. In addition, the use of non-overlapping samples each for the EFA and CFA allowed for the built-in replication of findings.

No nationally representative data were collected from any country. Data were collected from one site in each country, which limited diversity of the sample, with respect to some factors, such as caste, ethnicity, SES, etc. The role of intersectionality (i.e., the confluence of multiple social influences) cannot be ruled out, thus the results of this project may not be applicable to each individual within a particular nation or community. Second, the current study used data on the home environment from only one time point (24 months). Future analysis should examine the longitudinal nature of the home environment, including its stability and cross cultural differences over time. Future studies may also wish to look at how practices within cultures differed across groups, which may have contributed to the psychometric differences observed in the current study. Finally, the current study demonstrates the psychometric strengths of the HOME when used cross-culturally. Utilizing the HOME, further studies should extend the current findings to examine differences in how the home environment impacts specific outcomes across cultures.

## 5 Conclusions

The goal of the current study was to: (1) examine the cross-cultural validity of the HOME measure among the international MAL-ED sites, and (2) adapt an existing tool for evaluating the home environment. This is the first evaluation of the HOME measure utilizing measurement equivalence analytic techniques. The results suggest that the adapted HOME may be useful in evaluating the home environment across heterogeneous groups. Future studies should seek to replicate and extend these findings, particularly the relationship between the home environment and other variables associated with childhood developmental outcomes. Furthermore, a cross-culturally validated adaptation of the HOME could then also be used to evaluate cultural differences in the home environment, and how those differences are related to such factors as academic outcomes.

## Appendix A

### Bangladesh site

The Bangladeshi site is located in the capital city of Dhaka, Bauniabadh area of Mirpur, within an urban, squatter settlement (slum) with one of the highest population densities in the world. Dhaka has a tropical climate with high temperatures (temperatures range from 71^o^ F to 90^o^ F, with a mean of 64.4^o^ F, 18^o^ C) and high humidity. There are three distinct seasons: the pre-monsoon hot season; the rainy season, and a cooler, dry winter. The average family size is 4.5 with gross monthly income around $125. Overcrowding, poor house construction and poor sanitary disposals are common features of the community. Almost 63% families live in single roomed houses of metal and brick construction and 7.4% have mud or wooden floor. Around 99% of the households have access to supplied piped water in the dwellings or yard and 56% have latrine that flushes to septic tank. In regard to highest level of maternal education, 19% of mothers had completed no formal education, 47% had completed one to 5 years (primary school), 33% had completed six to 10 years, and 1% had completed secondary school or higher (> 10 years). The area has one UNICEF and one NGO operated pre-primary schools and around 25 Government and private high schools. The quality of pre-schools is limited due to large number of students. For more background and demographic information about the Bangladesh site, see Ahmed et al. (2014).

### Brazil site

The Brazil site is located in the city of Fortaleza, the fifth most populated city in Brazil. Fortaleza is located in the state of Ceará. The climate of Ceará is semi-arid with low levels of rainfall throughout the year and an average temperature of 83 °F (29 °C). In this sample, the average number of people living in each home was 5.2. In regard to highest level of maternal education completed, 8% of mothers completed primary school (one to 5 years), 34% completed secondary school (six to 10 years), and 60% completed secondary school or higher (> 10 years). In regard to employment, most individuals in this area report being involved in local labor. However, it is also known that many inhabitants are involved in the narcotics trade; thus, the accuracy of the reports is unclear. In the local community, there are a few small private and one public child care pre-school centers. For more background and demographic information about the Brazil site, see Lima et al. (2014).

### India site

The India site is located in Vellore, Tamil Nadu the southern region of India. The climate ranges from semi-humid to semiarid with three seasons: a southwest monsoon season, a northeast monsoon season, and a dry season with average temperatures ranging from 24 °C (74 °F) to 28 °C (83 °F) depending on the season. The average family size is 5.7. In the MAL-ED cohort at this site, the average education for the heads of household of MAL-ED families is 6.81 years (range 0 to 18 years). In regard to occupation, there is a high rate of unemployment at the India site (58.5%). For adults who are employed, most are involved in daily wage labor. For more background and demographic information about the India site, see John et al. (2014).

### Nepal site

The Nepal site is located in the Bhakatapur municipality which is approximately 15 km outside of the capital city of Nepal, Kathmandu. The climate in this region is subtropical with a wet and hot rainy season (May to August) as well as a cool and dry season (October to March) with an average temperature of 95 °F, 35 °C. The nation of Nepal is diverse in regard to ethnic and caste groups. The majority of the citizens of Bhakatapur are members of the Newar ethnic group. In regard to family demographics, most families live in extended family systems. The average family size is 4.8, and most families are employed in agriculture. In regard to maternal education, 94% of the mothers at this site were literate but only a quarter of the mothers had completed more than 10 years of formal education. For more background and demographic information about the Nepal site, see Shrestha et al. (2014).

### Pakistan site

The Pakistan site located in Naushahro Feroze which is a rural area in the Southern region of Pakistan. The summers are very hot (average temperature 113 °F, 45 °C). In regard to family demographics, most families live in extended family systems. The average number of children per family is 4 with an average family size of 6 to 7. Approximately 33% of mothers at this site area able to read, and the average maternal education is 2 years. Approximately 60–70% of fathers in this region are literate, and males tend to have greater access to educational opportunities. The primary occupation for men is farming and most women are homemakers or are involved in weaving and stitching. Approximately 60% of children are malnourished at 2 years of age. Children tend to start pre-school between the ages of 5 and 6. Schools in this region have highly limited resources. Some schools do not have electricity, lack basic materials, and have a high rate of teacher absenteeism. For more background and demographic information about the Pakistan site, see Turab et al. (2014).

### Peru site

The Peru site is located along a string of semi-urban communities bordering the River Nanay 14 km from in the rural city of Iquitos in the Loreto province. Loreto is located in a low tropical rainforest climate. The average temperature is 78 °F, annual rainfall is 2.6 m a year with April being the typical month where the river reaches its peak and when flooding is common. In regard to maternal education at this site there in near-universal literacy, 1.1% of mothers had no formal education or did not respond, 21.1% had one to 5 years of education, 58.2% had six to 10 years of education, 14.1% completed their secondary education (> 10 years), and 5.6% completed come college or technical school. Primary occupations for men in this region including growing and selling vegetables, fishing, moto-taxi driving, and brick-making. The majority of women are homemakers, but some are employed as shopkeepers, maids, or work in local small scale commerce. At this site, the majority (94–99%) of children over age 6 attend school. Approximately 32.5% of families in the area reported enrollment in the *JUNTOS* program which is a national program that provides cash transfer to families with children under age 14 who maintain 85% school attendance, obtain civic identification documentation, maintain regular health check-ups, and receive vaccinations. For more background and demographic information about the Peru site, see Yori et al. (2014).

### South Africa site

The South African site is located in the Dzimauili community within the Vhembe district in the Northern part of the Limpopo province. Overall, the Vhembe district has a warm, semi-arid climate. In regard to family demographics, mothers in the sample had an average of 2.4 live births. All participants in the sample reported their race as Black. On average, mothers in the sample had completed 9 years of formal education. Most participants from this site were engaged in subsistence farming, minor trading, or government agency work for more background and demographic information about the South African site, see Bessong, Nyathi, Mahopo, and Netshandama (2014).

### Tanzania site

The Tanzania site is located in Haydom which is a rural area, approximately 300 km from the city of Arusha, in the North-Central region of Tanzania. The North-Central region of Tanzania has three seasons: a high season which is cool and dry; a shoulder season which is hot with some rain; and a low season with heavy rains. The average temperature is 67.1 °F (19.5 °C), but temperatures vary by season. In this sample, the average woman had 4.3 live births and 1.3 child deaths and there were an average of 6.7 people living in each household. In regard to formal education, the mothers in this sample had completed 7 years of schooling on average; however, 15.3% of women in the sample had no formal schooling. Schools lack basic sanitary facilities and the teacher-pupil ratio is high, about 1 to 50. The area is populated by a diverse mix of agropastoralists, agriculturalist and pastoralists. For more background and demographic information about the Tanzania site, see Mduma et al. (2014).

### HOME observation items in MAL-ED

*Note*. Item stems have been altered slightly to protect copyrighted material.

1. Caregiver spontaneously vocalizes to the child during the visit.
2. Caregiver verbally responds to child's vocalizations.
3. Caregiver tells the child the name of some person or object during the visit.
4. Caregiver's speech is clear.
5. Caregiver initiates verbal exchanges with the observer.
6. Caregiver expresses ideas and is conversational (i.e., gives more than brief answers).
7. Caregiver spontaneously praises child twice during visit.
8. When speaking to the child, caregiver's voice conveys positive feeling.
9. Caregiver does something affectionate with the child (hugs, pats, etc.) during the visit.
10. Caregiver shows some positive emotional response to the child.
11. Caregiver smiles at or laughs with the child.
12. Caregiver does not shout at the child.
13. Caregiver does not overtly display annoyance or hostility toward the child.
14. Caregiver does not slap or spank the child.
15. Caregiver reports no physical punishment within the past week.
16. Caregiver does not scold/criticize the child during the visit.
17. Caregiver keeps the child within visual range and looks at the child frequently.
18. Caregiver talks to the child while doing chores.
19. Caregiver intentionally encourages developmental advances.
20. Caregiver provides structure in the child's day.
21. Caregiver believes the child's behavior can be changed or modified by the parent.
22. When the primary caregiver is away, care is provided by one of three regular others.
23. Child is not cared for by another child (under age 12).
24. There is no evidence that anyone handles the child inappropriately.
25. Someone brings the child to a shop or market at least once a week.
27. The child is taken to the health clinic regularly.
28. The child has a special place to keep his toys and things
29. The child's play area is relatively safe and free from hazards.
30. The stove is located in a relatively safe area.
31. The house is relatively light.
32. The house is relatively ventilated.
33. The house is relatively clean.
34. The house is relatively neat and orderly.
35. There are appropriate play materials (e.g., toys, tins, balls, dolls, slates) in the house
36. The child has a riding toy for gross motor stimulation.
37. The caregiver provides toys or activities during the visit.
38. There are some magazines, newspapers, or books visible in the home.
39. The family has a pet.
40. The father plays with the child or is involved with caregiving every day.
41. The caregiver tells the child stories or nursery rhythms at least weekly.
42. The caregiver sings to the child daily.
43. The child eats at least one meal per day with the caregiver and the father.
44. The family has not moved more than once in the past year.
45. The family visits with relatives at least once per month.
46. The family visits with close friends at least once per month.
47. The child is relatively clean, with no offensive odor.
48. The child's hair is relatively clean.
49. The child's clothes are relatively clean.

## Appendix B MAL-ED investigators and institutional affiliations

Angel Mendez Acosta^1^

Rosa Rios de Burga^1^

Cesar Banda Chavez^1^

Julian Torres Flores^1^

Maribel Paredes Olotegui^1^

Silvia Rengifo Pinedo^1^

Mery Siguas Salas^1^

Dixner Rengifo Trigoso^1^

Angel Orbe Vasquez^1^

Imran Ahmed^2^

Didar Alam^2^

Asad Ali^2^

Zulfiqar A Bhutta^2^

Shahida Qureshi^2^

Muneera Rasheed^2^

Sajid Soofi^2^

Ali Turab^2^

Anita KM Zaidi^2^

Ladaporn Bodhidatta^3^

Carl J Mason^3^

Sudhir Babji^4^

Anuradha Bose^4^

Ajila T George^4^

Dinesh Hariraju^4^

M Steffi Jennifer^4^

Sushil John^4^

Shiny Kaki^4^

Gagandeep Kang^4^

Priyadarshani Karunakaran^4^

Beena Koshy^4^

Robin P Lazarus^4^

Jayaprakash Muliyil^4^

Mohan Venkata Raghava^4^

Sophy Raju^4^

Anup Ramachandran^4^

Rakhi Ramadas^4^

Karthikeyan Ramanujam^4^

Anuradha Rose^4^

Reeba Roshan^4^

Srujan L Sharma^4^

Shanmuga Sundaram E^4^

Rahul J Thomas^4^

William K Pan^5,6^

Ramya Ambikapathi^6^

J Daniel Carreon^6^

Vivek Charu^6^

Viyada Doan^6^

Jhanelle Graham^6^

Christel Hoest^6^

Stacey Knobler^6^

Dennis R Lang^6, 7^

Benjamin JJ McCormick^6^

Monica McGrath^6^

Mark A Miller^6^

Archana Mohale^6^

Gaurvika Nayyar^6^

Stephanie Psaki^6^

Zeba Rasmussen^6^

Stephanie A Richard^6^

Jessica C Seidman^6^

Vivian Wang^6^

Rebecca Blank^7^

Michael Gottlieb^7^

Karen H Tountas^7^

Caroline Amour^8^

Eliwaza Bayyo^8^

Estomih R Mduma^8^

Regisiana Mvungi^8^

Rosemary Nshama^8^

John Pascal^8^

Buliga Mujaga Swema^8^

Ladislaus Yarrot^8^

Tahmeed Ahmed^9^

AM Shamsir Ahmed^9^

Rashidul Haque^9^

Iqbal Hossain^9^

Munirul Islam^9^

Mustafa Mahfuz^9^

Dinesh Mondal^9^

Fahmida Tofail^9^

Ram Krishna Chandyo^10^

Prakash Sunder Shrestha^10^

Rita Shrestha^10^

Manjeswori Ulak^10^

Aubrey Bauck^11^

Robert Black^11^

Laura Caulfield^11^

William Checkley^11,6^

Margaret N Kosek^11^

Gwenyth Lee^11^

Kerry Schulze^11^

Pablo Peñataro Yori^11^

Laura E Murray-Kolb^12^

A Catharine Ross^12^

Barbara Schaefer^12,6^

Suzanne Simons^12^

Laura Pendergast^13^

Cláudia B Abreu^14^

Hilda Costa^14^

Alessandra Di Moura^14^

José Quirino Filho^14,6^

Alexandre Havt^14^

Álvaro M Leite^14^

Aldo AM Lima^14^

Noélia L Lima^14^

Ila F Lima^14^

Bruna LL Maciel^14^

Pedro HQS Medeiros^14^

Milena Moraes^14^

Francisco S Mota^14^

Reinaldo B Oriá^14^

Josiane Quetz^14^

Alberto M Soares^14^

Rosa MS Mota^14^

Crystal L Patil^16^

Pascal Bessong^17^

Cloupas Mahopo^17^

Angelina Maphula^17^

Emanuel Nyathi^17^

Amidou Samie^17^

Leah Barrett^18^

Rebecca Dillingham^18^

Jean Gratz^18^

Richard L Guerrant^18^

Eric Houpt^18^

William A Petri, Jr^18^

James Platts-Mills^18^

Rebecca Scharf^18^

Binob Shrestha^19^

Sanjaya Kumar Shrestha^19^

Tor Strand^19,15^

Erling Svensen^20,8^

### Institutions

^1^A.B. PRISMA, Iquitos, Peru

^2^Aga Khan University, Karachi, Pakistan

^3^Armed Forces Research Institute of Medical Sciences, Bangkok, Thailand

^4^Christian Medical College, Vellore, India

^5^Duke University, Durham, NC, USA

^6^Fogarty International Center/National Institutes of Health, Bethesda, MD, USA

^7^Foundation for the NIH, Bethesda, MD, USA

^8^Haydom Lutheran Hospital, Haydom, Tanzania

^9^icddr,b, Dhaka, Bangladesh

^10^Institute of Medicine, Tribhuvan University, Kathmandu, Nepal

^11^Johns Hopkins University, Baltimore, MD, USA

^12^The Pennsylvania State University, University Park, PA, USA

^13^Temple University, Philadelphia, PA, USA

^14^Universidade Federal do Ceara, Fortaleza, Brazil

^15^University of Bergen, Norway

^16^University of Illinois at Chicago, IL, USA

^17^University of Venda, Thohoyandou, South Africa

^18^University of Virginia, Charlottesville, VA, USA

^19^Walter Reed/AFRIMS Research Unit, Kathmandu, Nepal

^20^Haukeland University Hospital, Bergen, Norway

## Footnotes

2 PE = parameter estimate
